# Individual and occupational risk factors for knee osteoarthritis – Study protocol of a case control study

**DOI:** 10.1186/1471-2474-9-26

**Published:** 2008-02-26

**Authors:** André Klußmann, Hansjuergen Gebhardt, Falk Liebers, Lars Victor von Engelhardt, Andreas Dávid, Bertil Bouillon, Monika A Rieger

**Affiliations:** 1 Institute of Occupational Health, Safety and Ergonomics (ASER) at the University of Wuppertal, Corneliusstrasse 31, 42329 Wuppertal, Germany; 2 Federal Institute for Occupational Safety and Health, Noeldnerstrasse 40-42, 10317 Berlin, Germany; 3 Department of Trauma and Orthopedic Surgery, University of Witten/Herdecke, HELIOS Hospital Wuppertal, Heusnerstr. 40, 42283 Wuppertal, Germany; 4 Department of Trauma and Orthopedic Surgery, University of Witten/Herdecke, Hospital Cologne Merheim, Ostmerheimerstr. 200, 51109 Cologne, Germany; 5 Department of Occupational Health and Environmental Medicine, Institute of General Practice and Family Medicine, University of Witten/Herdecke, Alfred-Herrhausen-Str. 50, 58448 Witten, Germany

## Abstract

**Background:**

Knee osteoarthritis (OA) is one of the frequent and functionally impairing disorders of the musculoskeletal system. In the literature, a number of occupational risk factors are discussed as being related to the development and progress of knee joint diseases, e.g. working in kneeling or squatting posture, lifting and carrying of heavy weights. The importance of the single risk factors and the possibility of prevention are currently under discussion. Besides the occupational factors, a number of individual risk factors are important, too. The distinction between work-related factors and individual factors is crucial in assessing the risk and in deriving preventive measures in occupational health. In existing studies, the occupational stress is determined mainly by surveys in employees and/or by making assumptions about individual occupations. Direct evaluation of occupational exposure has been performed only exceptionally.

The aim of the research project ArGon is the assessment of different occupational factors in relation to individual factors (e.g. constitutional factors, leisure time activities, sports), which might influence the development and/or progression of knee (OA). The project is designed as a case control study.

**Methods/Design:**

To raise valid data about the physical stress associated with occupational and leisure time activities, patients with and without knee OA are questioned by means of a standardised questionnaire and an interview. The required sample size was estimated to 800 cases and an equal number of controls. The degree and localisation of the knee cartilage or joint damages in the cases are documented on the basis of radiological, arthroscopic and/or operative findings in a patient record. Furthermore, occupational exposure is analysed at selected workplaces. To evaluate the answers provided in the questionnaire, work analysis is performed.

**Discussion:**

In this research project, specific information on the correlation of occupational and individual factors on the one hand and the current state of knee OA on the other will be analysed in order to describe preventive measures. In addition, information regarding a better evaluation of various forms of physical stress in different occupations will be available. This might lead to more effective prevention strategies.

## Background

### Definition of knee osteoarthritis

Osteoarthritis (OA) is the most common joint disorder, characterised by an imbalance between the synthesis and degradation of the articular cartilage, leading to the classic pathologic changes leading to destruction of cartilage [1]. The breakdown and deterioration of cartilage leads to the formation of new bone at the joint surfaces (sclerosis) and margins (osteophytes) [2]. This process often results in joint pain and loss of mobility, which may lead to long-term disability. Although OA is considered a non-inflammatory form of arthritis, there can be a small inflammatory component. Knee OA characterises all degenerative changes of the knee joint and is a disease with a multi-factorial aetiology. A primary and secondary form of knee OA may be differentiated [3]:

 primary (idiopathic) knee OA:

Osteoarthritis is classified as primary when aetiology and pathogenesis are unknown. The reasons for an endogenous cartilage formation defect are under discussion. Clinical manifestations start increasing from the age of 40, heredity has been described.

 secondary knee OA:

The causes are mechanical or metabolic risk factors such as aberrance of the axis, haemophilia, rheumatoid and bacterial arthritis, osteochondrosis dissecans, dysplasia of the joint, injury of the knee joint, etc. For patients with cartilage defects in their medical history, clinical studies demonstrate multiple risks for early onset of arthrosis [4]. Animal experiments indicate that even minor untreated cartilage injuries of a critical size greater than 5 mm may result in persistent damage of the joint [5,6]. People who repetitively stress one joint or group of joints (e.g. foundry workers or coal miners) are particularly at risk. Much of the risk of osteoarthritis of the knee comes from occupations that involve repetitive bending of the joint [7]. Obesity may be a further factor in the development of osteoarthritis, particularly of the knee and especially in women [8]. However, once osteoarthritis has developed, the work-related repetitive movement often makes the disorder worse.

**Figure 1:**
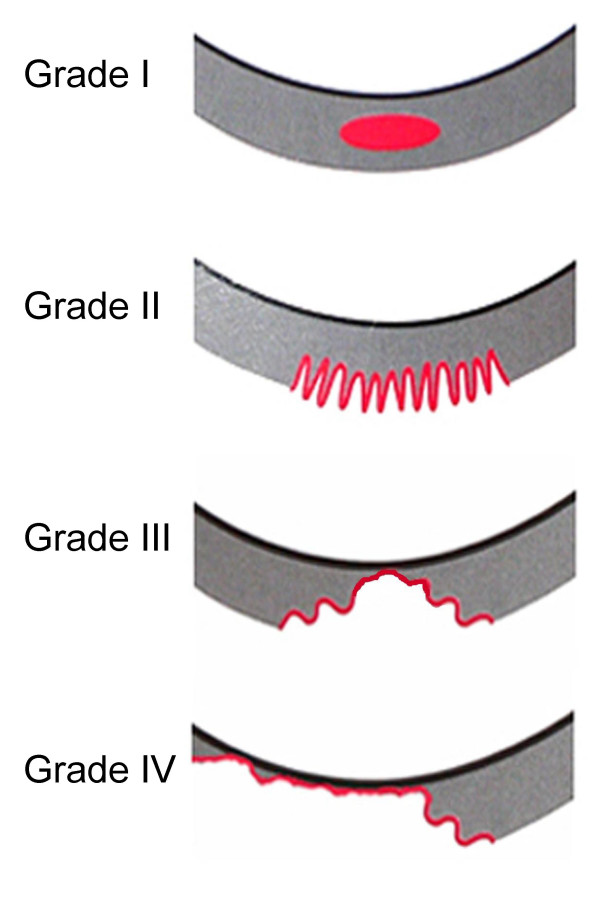
Schematic drawings to illustrate the grading of cartilage diseases as described by Outerbridge [9].

OA is normally classified in different grades. With regard to the knee, the use of a four-grade classification based on Outerbridge [9] is common for arthroscopic findings. Thus, the arthroscopic classification used in this study is defined as follows (Fig. 1):

 Grade I: Softening and swelling of the cartilage. No damage of cartilage surface.

 Grade II: Fragmentation and fissuring in an area half an inch or less in diameter. Only shallow damage of cartilage. Surface is fringed, shredded and resembles crab meat.

 Grade III: Fragmentation and fissuring in an area more than half an inch in diameter. Deep damage of cartilage down to bone, visible to the naked eye.

 Grade IV: Erosion of cartilage down to bone. Complete destruction of cartilage. Large damage of joint, cicatricial tissue, cartilage bald.

For the documentation of knee damage based on radiological findings, the four-grade classification of Kellgren and Lawrence [10] is commonly used. Thus, the radiological classification used in this study is defined as follows:

 Grade I: Doubtful narrowing of joint space and possible osteophytic lipping.

 Grade II: Definite osteophytes and possible narrowing of joint space.

 Grade III: Moderate multiple osteophytes, definite narrowing of joint space, some sclerosis and possible deformity of bone ends.

 Grade IV: Large osteophytes, marked narrowing of joint space, severe sclerosis and definite deformity of bone ends.

### Prevalence and findings regarding knee symptoms

The considered studies showed a prevalence of knee OA between 27–90% in people of 60 years or older [3]. Approximately 10–12% of all individuals suffer from cartilage lesions [11]. In a cross-sectional study among 1,000 employees doing mainly office work, a 12-month-prevalence of symptoms was reported between 22–28% with only slight differences in age groups (<25, <35, <45, <55 and 55–65 years). In the same study, employees who worked in the laboratory, storehouse or in production as well as in the office reported slightly increased values [12]. A survey in more than 200 employees in emergency medical services revealed an age-dependent 12-month prevalence between 17–35%. In this case, and in contrast to the previously mentioned study in office workers, the frequency of knee joint disorders correlated significantly with age. Moreover, the prevalence of disorders was partially associated with the high physical stress of lifting and carrying heavy loads (e.g. patient + stretcher) [13].

Several occupational risk factors such as working in kneeling or squatting posture, heavy lifting and carrying of weights are discussed as being related to the development and progress of knee joint diseases [14-20]. The diagnosis-related analysis of work incapacity days from a German health insurance database of 18.5 million employees revealed that a higher risk appeared for a number of occupations involving knee-straining activities [21]. As study results are not identical, the importance of specific risk factors of knee OA and the possibilities of prevention are currently under discussion. In Germany, so far several contradictory study results regarding the correlation between occupational stress factors and the appearance of knee joint diseases have been published [22-30]. The relevance of physical stress for initiating or aggravating knee OA is being controversially discussed [25]. However, besides the occupational risk factors, a number of individual factors (e.g. obesity, gender, leisure time behaviour, genetic disposition, metabolic syndrome, smoking behaviours or the regular practice of extreme sports [2,3,26]) may play a role. The distinction between work-related and individual factors is crucial for assessing the very risk and for deriving preventive measures in general and in the field of occupational health.

## Methods

### Aim of the study

With respect to the literature, occupational stress was mainly determined by surveys in employees or by making assumptions about individual jobs. Direct evaluation of occupational exposure has been performed only exceptionally in previous studies. Only little is known about the correlation between knee-straining occupational activities and the development of knee OA. Besides, studies involving dose depending effects are rare [16,19].

The aim of the research project "ArGon" (ArGon = "Arbeitsbedingungen" [working conditions] and "Gonarthrose" [knee OA]) is to determine the importance of occupational stress as a whole, of different single occupational factors (e.g. various kneeling and squatting activities, lifting and carrying of weights, standing, jumping), and other factors of influence (e.g. age, gender, constitutional factors) for the onset of knee OA.

### Research topics

#### Primary outcome

Possible effect of defined occupational tasks calculated as score on prevalence of knee OA.

#### Secondary outcome

1 Possible effect of relevant leisure time activities calculated as score on prevalence of knee OA.

2 Possible effect of relevant individual risk factors calculated as score on the prevalence of knee OA.

3 Possible effect of occupational and individual activities calculated as scores on the current state of knee damage (described in the patient records).

### Study design

This case control study is based on the population of two regions in Germany. The case group is recruited from the surgical-orthopaedic wards of university teaching hospitals and from appropriate outpatient clinics. Patients with documented knee OA fill out a standardised questionnaire during their hospitalisation. A standardised document with the clinical, radiological and arthroscopic findings is filled out by the physician treating the patient. This case group is compared with a control group to which the same questionnaire is submitted. The controls are recruited from the accident surgery services and matched with the case group according to age (± 5 years), gender, and place of living. The questionnaires are collected and evaluated in the study centre. In both groups, participants with jobs involving kneeling or lifting and carrying of heavy loads are interviewed in order to get additional, accurate information about the individual's work tasks.

Besides the consecutive recruitment of cases and controls in the hospitals, patients which could not be addressed directly during their hospitalisation are contacted in retrospect by the hospital physician.

### Instruments

#### Standardised questionnaire

This questionnaire contains questions about sociodemographic factors, musculoskeletal symptoms, duration of sitting, standing, moving and knee-straining work, frequency of lifting or carrying heavy loads, jumping, climbing steps per day as well as additional working conditions such as climate, time pressure etc. for all occupational employment and housework. Further questions pertain to disease & injuries, drinking alcohol, smoking, frequency and duration of sports, frequency and duration of various leisure activities, the individual's hormone balance, and current condition of the knees.

→ This questionnaire is filled out by all participants of the study.

#### Partially standardised telephone interview

This interview contains detailed questions on frequency and duration of e.g. kneeling, squatting, lifting and carrying for every occupational employment.

→ This interview will be conducted if any knee-straining work or any lifting or carrying of heavy loads is mentioned in the questionnaire or if any general information in the questionnaire is missing. A validation of the questionnaire is done in interviewing and observing employees within exemplary workplace in selected companies of the region.

#### Patient record

**Figure 2:**
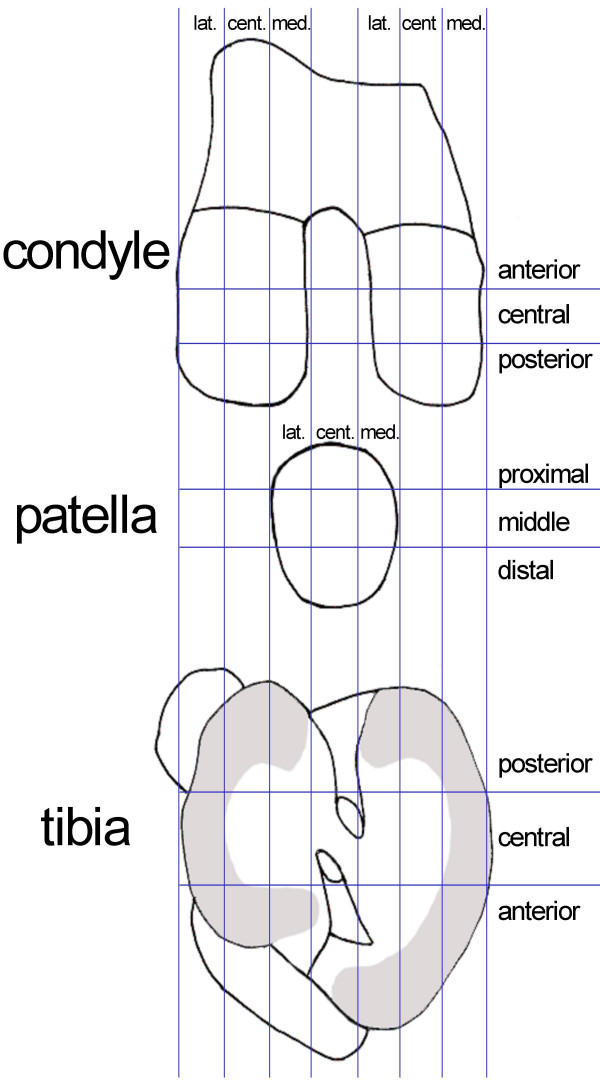
Standardised mapping of depth, degree and localisation of the cartilage damage according to the ICRS mapping method [31].

The patients' history and the physicians' findings are documented in a patient record including information on the general health status, condition of knee cartilage, meniscus and ligaments. In additional, depth, degree, and localisation of the cartilage damage is mapped (Fig. 2). The method of mapping is derived from the mapping method employed by the International Cartilage Repair Society (ICRS) [31].

### Sample size

In major systematic reviews essential studies of lower or upper limb disease are listed [32,33]. Previous case control studies involved usually not more than 500 cases and an equal number of controls.

In this case control study the correlation between knee OA on the one hand and occupational and non-occupational factors on the other will be estimated. It is already known from previous literature and practical experience that there is a wide range of influencing factors. Due to this and the many different occupations, 800 cases and an equal number of controls were estimated with EpiManager [34] as being necessary in order to achieve precise and statistically significant results.

Due to the various influencing factors, a power evaluation is difficult. Proof of effect (odds ratio (OR) of > 2) for frequent professional risk factors should be possible considering the size of the case and control groups. For example, a significantly higher prevalence of an occupational risk factor (OR>2) will be detected in the sample of 800 cases and controls with a power of 80% and α = 0.5 (two sided) if the prevalence is at least 4% among cases and 2% among controls and there are no confounding factors. As the prevalence of the some risk factors will be even higher, a power of 80% should be secure if confounding factors were to be considered. The same is true for statistical analyses performed in subgroups, such as gender-specific analysis for example. Thus, for most calculations the power of the data set can be considered sufficient.

### Recruitment and inclusion criteria of cases and controls

Cases are recruited from the surgical-orthopaedic wards of maximum care hospitals and from appropriate outpatient clinics; controls are recruited from the accident surgery services of the participating hospitals.

#### Case group

Only patients with a confirmed diagnosis of a clinically relevant knee OA in at least one knee are included. Clinical relevance is defined as having lead to any medical treatment, e.g. for pain reduce. The patient's history, physical examination, radiological examination, and arthroscopy are performed by an orthopaedic surgeon. The onset of symptoms is determined by anamnesis. Additional inclusion criteria are:

 age between 25 and 75 years

 treatment for knee disorders in one of the participating hospitals

 knee OA confirmed by means of at least one of the following diagnostics:

○ radiological: ≥ grade II on KELLGREN & LAWRENCE scale [10]

○ arthroscopy or open findings: ≥ grade III on OUTERBRIDGE scale [9]

 clinically relevant ongoing symptoms for no longer than 10 years

 place of residence in the vicinity of the participating hospitals

 linguistic and cognitive ability to understand and fill out the questionnaire and to give informed consent.

The exclusion criteria for cases are:

 non-fulfilment of the afore-listed criteria

 previous fractures involving knee joints or injuries of the knee (ligament or cartilage injuries)

 inflammatory or reactive knee joint illnesses

#### Control group

Patients treated in the accident surgery services of one of the participating hospitals are included in the control group if they fulfil the following criteria:

 age between 25 and 75 years

 treatment for an accident due to an external cause (i.e. not due to circulatory, metabolic or neurological disorders) The accident must not be work-related.

 place of residence in the vicinity of the participating hospitals.

 linguistic and cognitive ability to understand and fill out the questionnaire and to give informed consent.

The exclusion criteria for hospital controls are:

 non-fulfilment of the afore-listed criteria

 a physician has already diagnosed knee OA

Patients are rejected as control subjects if their accident has no external cause or if they had accident injuries and/or operations on the knee. Appropriate filter questions are used from a common questionnaire [35]: Have you already been operated on the knee? Has your physician already diagnosed knee OA? The definition "accident from external cause" was chosen in order to exclude secondary disorders which can lead, e.g., to a syncopation.

### Selection of the control group

In Germany, many research groups make use of the database of the public registry office of individual cities in order to recruit population-based controls. This office is a government agency in which all residents of a city or area are listed, including address and date of birth. With this database, a nearly unbiased sample of a defined region can be obtained. However, this method of recruitment can also be disadvantageous as response rates often turn out to be very low [36]. In addition, controls may return only incomplete questionnaires as their motivation for participation may be lower than in the case group. Thus data from controls may be not only not representative for the general population but also less informative than those of the case group.

To overcome these problems, hospital patients from the accident surgery services will be addressed as controls. During the hospital stay, the patients can be personally contacted by their treating physician. This condition may lead to an essentially higher response rate and higher quality of data than in controls from the public registry database. In addition, the setting for recruitment is the same for both cases and controls. This is important as similarity between recruiting cases and controls is the most important factor [37].

The degree to which the hospital control sample is representative for the general population has to be proven with respect to occupation and general health status. Population-based statistics (e.g. occupation, gender, age, health status) can be assessed with the help of databases of the regional branch of the Federal Statistics Office. Furthermore, the degree to which the health status can be considered representative can also be verified by comparing this information with other databases generated e.g. by the "Federal Health Survey 1998" (BGS '98 [38]) or other studies run simultaneously in the same geographical region by other research institutes.

The response rate and general health status of cases and controls addressed consecutively and in retrospect will be compared in order to control for any bias with respect to recruitment strategy.

### Data collection

All patients with knee OA being treated at one of the participating hospitals are checked for fulfilment of the inclusion criteria. If the patient fulfils all criteria, he/she is asked to participate and – after obtaining informed consent – is given a questionnaire. All patients are listed in a study log. In addition the physician treating the patient documents the medical history and maps pathological findings in the ICRS related scheme.

After approximately 14 days all data is transmitted to the study centre. The questionnaires and findings are then reviewed with respect to completeness and validity of data. Based on complete and correct data, the hospitals are instructed as to which controls are needed, so as to match the case group according to age (± 5 years), gender, and vicinity to hospital. When checking the questionnaires, those participants with jobs involving kneeling or lifting and carrying heavy loads are separately considered. These persons are called by telephone and interviewed a second time in order to get more accurate and detailed information about their work tasks.

### Content of method inventory and outcome parameters

The questionnaire used in the survey was derived partly from published instruments (Table 1).

**Table 1:** Content of method inventory and outcome parameters

**Parameter**	**Intention**	**Outcome**	Source*
sociodemographic factors	matching variables and basic description	distribution	[39]
musculoskeletal symptoms	description of health status	health status	[40]
frequency and duration of e.g. kneeling, squatting, lifting and carrying	occupational exposure	cumulative effects	[35, 41, 42 and own development]
diseases & injuries, drinking alcohol, smoking	possible confounding factors	cumulative effects	[35, 41, 42 and own development]
frequency and duration of sports	possible confounding factors	cumulative effects	
frequency and duration of leisure time activities	possible confounding factors	cumulative effects	
questions on hormone balance/imbalance	possible confounding factors	cumulative effects	[41]
questions on knee status	selection variables		[35]
knee status: grade & localities of knee OA, secondary disorders and kind of diagnostics	to be compared with occupational tasks		[own development]

* [35, 41, 42] cite research articles describing different studies and results. The use of the very items in the ArGon questionnaire was kindly permitted by the responsible authors on request of the study group.

### Quality control and assurance

The use of standardised and – if available and appropriate – already validated and/or evaluated instruments ensures high quality of work. The project is supported by an advisory board so as to guarantee optimal process and content quality. A standardised procedure is guaranteed by the training of the physicians who deal with the data and decide on in- or excluding of the patients.

### Timeframe of the study

The study team performed a pre-test in the participating hospitals in 2006. Data collection with the revised method inventory started in early 2007 and will presumably end in 2008.

### Description of risks

To our knowledge, neither serious risks nor undesired effects can arise out of completing the questionnaires. Nothing to this effect has been reported in the literature consulted. There are no specific risks related to the study.

### Ethical principles

The study has been planned and conducted in accordance with the German medical professional code and the Helsinki Declaration of 1996 as well as the German Federal Data Protection Act. The study protocol and its amendment were approved by the Ethics Committee of the University of Witten/Herdecke (approval no. 61/2006). The study was started after the Ethics Committee gave its written and unrestricted approval.

Patients participate in the study voluntarily. They are informed that they can cancel their participation at any time without reason and without negative consequences for their current or future medical care.

### Informed consent

Informed consent for participation is obtained before the survey. Patients receive written and spoken information about the main features of the study; i.e. about potential benefits for their health and their contribution to the common public welfare. If they accept the conditions of the study and their participation, they document their consent with their signature. A copy of this statement is intended to be kept by the patient for later reference or cancellation of participation. In addition, the consent for being contacted by phone is obtained with the informed consent for inclusion in the study.

In the event of study discontinuation, all data will be deleted, unless the patient explicitly wishes and affirms further analysis of his/her data.

### Data security/disclosure of original documents

All original documents are secured by the medical confidentiality regulations of the participating hospitals and are treated according to German Federal Data Protection Act. All study-related data and documents are stored on a protected central server of the study centre. Only selected members of the study team have access to the respective files.

## Discussion

In this research project, specific information on the correlation between occupational and individual factors regarding the current state of the knee OA (degree and locality) are expected. In addition, information towards a better classification of occupational hazards with regard to this chronic disease will be available in future, which might lead to more specific prevention strategies.

## Competing interests

The author(s) declare that they have no competing interests.

## Authors' contributions

AK, HG, MAR, and BB conceived and designed the study. In addition, LVvE and AD are involved in the execution of the study and the writing of this manuscript. FL represents the funding body, initiated this study and was closely involved in the planning and development of the study design. All authors read and approved the final manuscript.

## Pre-publication history

The pre-publication history for this paper can be accessed here:


